# Association between yearlong air pollution and moderate-severe atopic dermatitis: A United States cross-sectional claims analysis

**DOI:** 10.1016/j.jdin.2023.04.017

**Published:** 2023-07-06

**Authors:** Pavin Trinh, Johan A.C. Allerup, Shufeng Li, Justin Ko, Jennifer Chen, Eleni Linos, Albert S. Chiou

**Affiliations:** a Department of Dermatology, Stanford University School of Medicine, Stanford, California; b Department of Urology, Stanford University School of Medicine, Stanford, California

**Keywords:** air pollution, atopic dermatitis, epidemiology, moderate-severe atopic dermatitis, PM2.5, population health

*To the Editor:* Atopic dermatitis (AD) is a chronic, pruritic inflammatory skin disease affecting over 7% of US adults, with 40% having moderate-severe disease.^1^ Air pollution, specifically particulate matter ≤2.5 μm (PM_2.5_), has been associated with AD development among healthy individuals.^2^ In regional studies, short-term air pollution greatly exceeding Environmental Protection Agency (EPA) standards were associated with flaring of AD symptoms.^3^ However, most US individuals chronically live at PM_2.5_ levels within EPA standards,^4^ and it is unclear how yearlong pollution at these regulated levels affects AD severity nationwide.

To investigate the association between yearlong PM_2.5_ pollution and AD severity among AD patients, we performed a cross-sectional study of AD patients between October 19, 2017 and October 19, 2018, using the Clinformatics Data Mart Database, a de-identified national insurance claims dataset. Patients at least 12 years old with at least 6 months of continuous enrollment, an AD ICD-10 code, and either an asthma or allergic rhinitis code were included (Supplementary Table I, available via Mendeley at https://doi.org/10.17632/zsyrpbmx6j.1).^5^ The primary outcome of having moderate-severe AD was identified by receipt of dupilumab, phototherapy, or a systemic immunosuppressant. Yearlong average PM_2.5_ levels were collected from EPA stations nearest to each patient’s zip code. Categorical and continuous variables were compared using Chi-squared and Student’s t-tests, respectively. Adjusting for age, sex, insurance, and urbanization, multivariate logistic regression calculated odds ratios for associations between yearlong average PM_2.5_ levels and moderate-severe AD. *P*-values < .05 were considered statistically significant.

Our study included 31,405 AD patients with a mean (SD) age of 46 (22) years and 64% female (Table I). Moderate-severe AD patients were more likely to be older (48 vs 46) and live in nonmetropolitan areas (90% vs 92%). Annual average PM_2.5_ ranged from 1.56 to 21.82 μg/m^3^. Mean yearlong average PM_2.5_ (8.35 vs 8.39 μg/m^3^) was comparable between moderate-severe and mild AD patients. No statistically significant associations existed between yearlong PM_2.5_ (OR = 1.001; 95% CI, 0.987-1.015, *P* = .905) concentrations and AD severity (Table II).

**Table I:** Demographic characteristics of patient cohort

	Total	Mild AD	Moderate-severe AD	*P*-value
	*N* = 31,405	*N* = 22,317	*N* = 9088	
Sex, % female (No.)	64% (20,062)	64% (14,191)	65% (5871)	.090
Age, mean (SD), y	46 (22)	46 (23)	48 (21)	<.001
Age group, % (No.), y				<.001
12-17	15% (4863)	17% (3883)	11% (980)	
18-34	20% (6224)	20% (4403)	20% (1821)	
35-44	12% (3615)	11% (2540)	12% (1075)	
45-54	12% (3849)	12% (2593)	14% (1256)	
55-64	12% (3843)	11% (2540)	14% (1303)	
65-74	18% (5511)	17% (3832)	18% (1679)	
75 and older	11% (3500)	11% (2526)	11% (974)	
Insurance, % (No.)				<.001
HMO	16% (5107)	16% (3554)	17% (1553)	
EPO	8% (2540)	8% (1842)	8% (698)	
POS	52% (16,361)	53% (11,738)	51% (4623)	
PPO	3% (1077)	3% (709)	4% (368)	
Other	20% (6320)	20% (4474)	20% (1846)	
Urbanization, % (No.)				.004
Rural	1% (402)	1% (273)	1% (129)	
Small town	2% (730)	2% (490)	3% (240)	
Micropolitan	5% (1620)	5% (1112)	6% (508)	
Metropolitan	91% (28,653)	92% (20,442)	90% (8211)	
PM2.5, mean (SD), ug/mˆ3	8.38 (1.85)	8.39 (1.85)	8.35 (1.82)	.067
PM10, mean (SD), ug/mˆ3	19.21 (6.40)	19.22 (6.42)	19.16 (6.35)	.448
SO2, mean (SD), ppb	1.83 (3.20)	1.81 (3.14)	1.88 (3.33)	.062
CO, mean (SD), ppm	0.43 (0.17)	0.43 (0.17)	0.43 (0.17)	.570
NO2, mean (SD), ppb	21.62 (9.27)	21.78 (9.27)	21.21 (9.25)	<.001
O3, mean (SD), ppb	39.56 (5.05)	39.48 (5.13)	39.74 (4.83)	<.001

*AD*, Atopic dermatitis; *CO*, carbon monoxide; *EPO*, exclusive provider organization; *HMO*, health maintenance organization; *NO2*, nitrogen dioxide; *O3*, ozone; *PM2.5*, fine particulate matter (<2.5 μm in diameter); *PM10*, coarse particulate matter (2.5-10.0 μm in diameter); *POS*, point of service; *PPO*, preferred provider organization; *SO2*, sulfur dioxide.

**Table II:** Multivariate logistic regression - Association between environmental/demographic factors and moderate-severe AD

	Odds ratio	95% CI	*P*-value
PM2.5, ug/mˆ3	1.001	0.987-1.015	.905
PM10, ug/mˆ3	0.998	0.993-1.003	.400
SO2, ppb	1.002	0.995-1.010	.577
CO, ppm	1.103	0.947-1.286	.209
NO2, ppb	0.991	0.988-0.995	<.001
O3, ppb	1.014	1.008-1.019	<.001
Temperature, F	1.001	0.998-1.004	.468
Female	1.037	0.985-1.091	.163
Age	1.007	1.005-1.008	<.001
Urbanization			
Rural	1.000	-	-
Small town	1.055	0.812-1.370	.688
Micropolitan	0.982	0.776-1.242	.878
Metropolitan	0.941	0.759-1.166	.578
Insurance			
HMO	1.000	-	-
EPO	0.966	0.864-1.081	.546
POS	1.023	0.947-1.104	.568
PPO	1.089	0.944-1.256	.244
Other	0.823	0.755-0.898	<.001

*AD*, Atopic dermatitis; *CO*, carbon monoxide; *EPO*, exclusive provider organization; *HMO*, health maintenance organization; *NO2*, nitrogen dioxide; *O3*, ozone; *PM10*, coarse particulate matter (2.5-10.0 μm in diameter); *PM2.5*, fine particulate matter (<2.5 μm in diameter); *POS*, point of service; *ppb*, parts per billion; *ppm*, parts per million; *PPO*, preferred provider organization; *SO2*, sulfur dioxide.

With increasing frequency of wildfires in the setting of climate change, there is concern over the impact of worsening pollution on conditions like AD. Balanced against this; however, are the sustained gains in air quality. Over the past 20 years, annual US average PM_2.5_ concentrations declined by 37% to 8.47 μg/m^3^, well below the EPA’s standard of 12 μg/m^3^.^4^ Our study found that most AD patients in the US live within this standard, with a mean annual average PM_2.5_ concentration of 8.38 μg/m^3^ and the 95th percentile experiencing an annual average of 10.89 μg/m^3^. PM_2.5_ has only been associated with AD development or increased health care use for AD when average long-term PM_2.5_ levels exceeded 24.78 μg/m^3^ or when short-term PM_2.5_ increased above 80 μg/m^3^, respectively.^2^^,^^3^ This study is limited by its reliance on ICD codes, patient’s zip codes during the study period, and patients with commercial insurance or Medicare Advantage. Overall, for AD patients living within EPA standards, yearlong PM_2.5_ was not associated with AD severity. While a chronic air pollution threshold that exacerbates AD may exist, it does not appear to fall within ranges typically experienced in the US at this time.

## Conflicts of interest

A.S.C. has served as a clinical investigator and/or consultant for: Regeneron Pharmaceuticals, Sanofi, AbbVie, Dermira, Eli Lilly, Pfizer. All other authors have no conflicts of interest, including specific financial interests and relationships and affiliations relevant to the subject of this manuscript, to disclose.
